# Supplementary material for: Functional annotation of creeping bentgrass protein sequences based on convolutional neural network
Source: BMC Plant Biol. 2022 May 2;22:227. doi: 10.1186/s12870-022-03607-8 (PMC9063134; doi:10.1186/s12870-022-03607-8)
Supplement: Supplementary file 1 — Additional file 1. [file 12870_2022_3607_MOESM1_ESM.docx]

**Additional file 1: Model’s performance with GO terms in three domains**

When establishing the prediction model, we trained the prediction model by the protein sequences collected from the Uniport database in the GO terms from three domains. To evaluate the performance of the model, we introduce five widely-used measurements, sensitivity $SE=\mathrm{TP}/(TP+FN)$, specificity $SP=TN/(TN+FP)$, precision $PR=TP/(TP+FP)$, accuracy $AC=(TP+TN)/(TP+TN+FP+FN)$，and Matthews correlation coefficient $MCC=(TP \times TN-FP\times FN)/$ $\sqrt{(TP+FP)(TP+FN)(TN+FP)(TN+FN)}$. Here T and F are for correct and wrong predictions, and N and P are for sequences labeled as positive and negative within the current model, respectively. For example, the FN is the number of sequences was wrongly labeled as negative (they are supposed to be positive). The training results for the selected GO terms are listed in Table S1 to show the performance of the prediction model, including the cellar component (CC) domain, molecular function (MF) domain and biological process (BP) domain.

**Table S1.**

The performance of the prediction model for the GO terms in the CC, MF and BP domains. The GO ID, term, and number N_GO_ of protein sequences in the GO terms are listed in the second to fourth columns，repectively. The measurements are listed in last five columns.

| Domain | GO ID | Function | N_GO_ | SE(%) | SP(%) | PR(%) | AC(%) | MCC |
| --- | --- | --- | --- | --- | --- | --- | --- | --- |
| CC | GO:0045202 | synapse | 9048 | 84.2 | 94.8 | 84.5 | 92.1 | 0.79 |
|  | GO:0055044 | symplast | 7369 | 93.5 | 99.8 | 99.2 | 98.2 | 0.95 |
|  | GO:0009295 | nucleoid | 1059 | 80.0 | 99.8 | 99.4 | 94.7 | 0.86 |
|  | GO:0031012 | extracellular matrix | 4429 | 68.5 | 98.9 | 95.5 | 90.9 | 0.76 |
|  | GO:0005576 | extracellular region | 36198 | 82.4 | 95.0 | 84.7 | 91.9 | 0.78 |
|  | GO:0030054 | cell junction | 20421 | 81.7 | 97.1 | 90.2 | 93.2 | 0.81 |
|  | GO:0031974 | membrane-enclosed lumen | 83898 | 78.0 | 95.5 | 85.1 | 91.2 | 0.76 |
| MF | GO:0005085 | guanyl-nucleotide exchange factor activity | 3070 | 72.4 | 98.8 | 95.1 | 92.5 | 0.79 |
|  | GO:0016247 | channel regulator activity | 1137 | 81.9 | 94.2 | 83.3 | 91.0 | 0.76 |
|  | GO:0005198 | structural molecule activity | 24949 | 85.2 | 99.2 | 97.3 | 95.6 | 0.88 |
|  | GO:0005215 | transporter activity | 59697 | 81.7 | 96.9 | 89.6 | 93.1 | 0.81 |
|  | GO:0016530 | metallochaperone activity | 328 | 88.9 | 100.0 | 100.0 | 97.7 | 0.93 |
|  | GO:0030545 | receptor regulator activity | 5048 | 86.6 | 97.6 | 92.6 | 94.8 | 0.86 |
|  | GO:0003700 | DNA-binding transcription factor activity | 50104 | 76.7 | 99.6 | 98.6 | 93.8 | 0.83 |
|  | GO:0060089 | molecular transducer activity | 31235 | 67.2 | 97.3 | 89.4 | 89.6 | 0.71 |
|  | GO:0016209 | antioxidant activity | 7154 | 88.1 | 99.4 | 98.1 | 96.6 | 0.91 |
|  | GO:0030234 | enzyme regulator activity | 25847 | 74.0 | 98.5 | 94.4 | 92.4 | 0.79 |
| BP | GO:0007610 | Behavior | 8061 | 75.6 | 98.2 | 93.2 | 92.7 | 0.80 |
|  | GO:0032502 | developmental process | 50081 | 34.8 | 97.1 | 80.1 | 81.6 | 0.44 |
|  | GO:0022610 | biological adhesion | 2225 | 67.6 | 96.6 | 86.9 | 89.3 | 0.70 |
|  | GO:0032501 | Multicellular organismal | 20389 | 50.1 | 94.0 | 73.1 | 83.2 | 0.51 |
|  | GO:0048518 | positive regulation of biological process | 63420 | 45.7 | 95.6 | 77.5 | 83.2 | 0.50 |
|  | GO:0040011 | locomotion | 11235 | 68.4 | 92.9 | 76.5 | 86.8 | 0.64 |
|  | GO:0001906 | cell killing | 938 | 56.7 | 98.8 | 93.8 | 88.3 | 0.67 |
|  | GO:0048519 | negative regulation of biological process | 53299 | 51.4 | 92.9 | 70.2 | 82.8 | 0.50 |
|  | GO:0051704 | multi-organism process | 5508 | 59.1 | 97.6 | 88.9 | 88.2 | 0.66 |
|  | GO:0022414 | reproductive process | 14011 | 43.2 | 97.5 | 85.0 | 84.0 | 0.53 |
|  | GO:0040007 | growth | 4586 | 60.6 | 99.0 | 95.3 | 89.6 | 0.71 |
|  | GO:0002376 | immune system process | 17317 | 53.2 | 97.2 | 86.2 | 86.4 | 0.60 |

For the CC domain, seven GO terms were considered as listed in Table 1. Since our aim is to establish a prediction model to annotate protein sequence with certain function from non-annotated data set correctly for further study, the most important measurement is the SP, which evaluates the method’s false positive control rate. For the GO terms considered here in CC domain, SP values are almost larger than 95%. Some of them are even larger than 99%, such as GO:0055044 and GO:0009295. It means the established model can correctly recognize the protein sequences in negative data set to ensure the correctness of final suggested sequences. The SE accesses the rate of recognized sequences from all sequences in a GO number. The values are in a range from 68 to 94%, which is obviously lower than SP. It means that some sequences will be missed by the trained model, which is the sacrifice for high SP. The results also suggest high precision (PR) and accuracy (AC). The MCC vales are also large for all GO terms considered, which reflects high correlation between a predictive value and the actual value. The results for the GO terms in the MF domain are also present in Table 1. The SP values are still considerable large. Most of them are larger than 95%, especially up to 100% of DO:0016530. The SE values are relatively small as same as in the CC domain. The values of PR and AC are also very large, most of which are larger than 90%. The MCC values are also larger than 0.7. For the BP domain, the MCCs are obviously smaller than above two domains, but still about 0.5 or larger. Most values of SP are larger than 95%, such as 99% of GO:0040007, which indicates high false positive control rate. The SE values are smaller, about 50%, which suggests more sequences missing compared with above two domains.
